# CCPlotR: an R package for the visualization of cell–cell interactions

**DOI:** 10.1093/bioadv/vbad130

**Published:** 2023-09-20

**Authors:** Sarah Ennis, Pilib Ó Broin, Eva Szegezdi

**Affiliations:** The SFI Centre for Research Training in Genomics Data Science, Galway, H91 TK33, Ireland; Discipline of Bioinformatics, School of Mathematical & Statistical Sciences, University of Galway, Galway, H91 TK33, Ireland; Apoptosis Research Centre, School of Biological & Chemical Sciences, University of Galway, Galway, H91 TK33, Ireland; The SFI Centre for Research Training in Genomics Data Science, Galway, H91 TK33, Ireland; Discipline of Bioinformatics, School of Mathematical & Statistical Sciences, University of Galway, Galway, H91 TK33, Ireland; The SFI Centre for Research Training in Genomics Data Science, Galway, H91 TK33, Ireland; Apoptosis Research Centre, School of Biological & Chemical Sciences, University of Galway, Galway, H91 TK33, Ireland

## Abstract

**Summary:**

We present CCPlotR—an R package that generates visualizations of cell–cell interactions. CCPlotR is designed to work with the output of tools that predict cell–cell interactions from single-cell gene expression data and requires only a table of predicted interactions as input. The package can generate a comprehensive set of publication-ready figures such as heatmaps, dotplots, circos plots and network diagrams, providing a useful resource for researchers working on cell–cell interactions.

**Availability and implementation:**

CCPlotR is available to download and install from GitHub (https://github.com/Sarah145/CCPlotR) and comes with a toy dataset to demonstrate the different functions. Support for users will be provided via the GitHub issues tracker (https://github.com/Sarah145/CCPlotR/issues).

## 1 Introduction

The increasing availability of single-cell RNA-sequencing data in recent years has led to the development of multiple tools for predicting cellular crosstalk (Armingol *et al.* 2021). These tools typically work by analysing the expression of genes that code for ligands and receptors from pairs of cell types that are known to interact with each other. Most tools will return a table of predicted interactions (Table 1) depicting the ligand, receptor, sending, and receiving cell types for each interaction, as well as a score to rank important interactions (e.g. specificity or expression score). Some tools also generate plots to visualize the predicted interactions but these are not consistent across tools and since most datasets include several cell populations, visualization of interactomes or specific features of interactions can be challenging.

**Table 1.** Example of input data for CCPlotR.

Source	Target	Ligand	Receptor	Score
B	CD8 T	HLA-DRA	LAG3	5.59
NK	NK	SPON2	ITGB2	4.22
CD8 T	B	LTB	CD40	3.87
B	NK	LGALS9	CD47	2.36

While reviews have been published which showcase different types of plots and reference the built-in visualization options included in some cell-cell interaction prediction tools, it can be time-consuming and impractical to install many of these tools just for visualization purposes (Almet *et al.* 2021, Armingol *et al.* 2021). This prompted us to develop CCPlotR—an R package that allows the user to easily generate cell–cell interaction plots using minimal code, regardless of which tool was used to predict the interactions.

## 2 The CCPlotR R package

### 2.1 Input

CCPlotR requires only a table of predicted interactions as input (see Table 1 for an example), with a column depicting the sending cell type, receiver cell type, ligand, receptor, and score for each interaction. The package is designed to work with the output of tools that predict cell–cell interactions from single-cell gene expression data, such as CellPhoneDB (Efremova *et al.* 2020), Liana (Dimitrov *et al.* 2022), NATMI (Hou *et al.* 2020), CellChat (Jin *et al.* 2021) etc. However, the plots are generic and could also be used to visualize cell–cell interactions predicted from bulk gene expression or proteomic data of pure cell populations as long as the input contains the same columns as Table 1. For example CCCExplorer (Choi *et al.* 2015), CellPhoneDB (Efremova *et al.* 2020), and ICELLNET (Noël *et al.* 2021) indicate in their documentation that they may be applied to bulk data provided the bulk transcriptomes are separate.

### 2.2 Plotting functions

**Figure 1.**
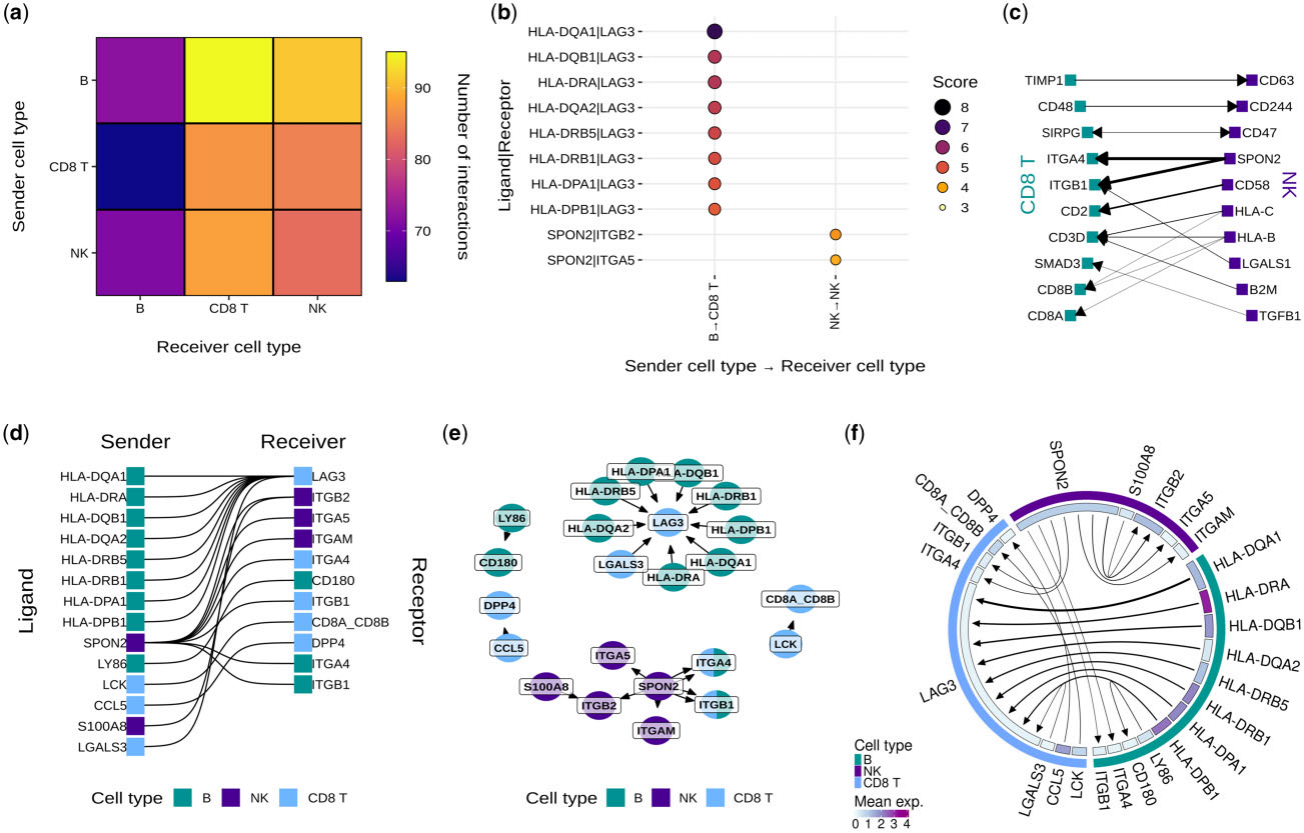
Examples of the six plotting functions in CCPlotR. (a) Plot generated with cc_heatmap showing the total number of interactions between each pair of cell types in the toy dataset. (b) Plot generated with cc_dotplot showing the top 10 interactions in the toy dataset. (c) Plot generated with cc_arrow showing the top 15 interactions between CD8 T and NK cells in the toy dataset. The weight of the arrows depicts the score for the interaction. (d–f) Plots generated with cc_sigmoid (d), cc_network (e), and cc_circos (f) showing the top 20 interactions in the toy dataset.

CCPlotR contains six different plotting functions that can generate several types of plots such as heatmaps, dotplots, circos plots, and network diagrams (Fig. 1). Each of the plotting functions contain options to generate different subtypes of plots (e.g. option A, B, C, etc.). The subtypes of plots fall into three main categories depending on what type of information they display:

1. Plots that show the number of interactions between different pairs of cell types (Fig. 1a).
2. Plots that show the interacting ligands and receptors as well as which cell types are expressing them (Fig. 1b–e).
3. Plots that show the interacting ligands and receptors and cell types as well as the expression levels of the ligand and receptor genes in each cell type (Fig. 1f).

For plots in category 3 that also show the expression levels of the ligand and receptor genes, the user is required to supply a second table which contains the average expression of each gene in each cell type.

The wide range of plot types included in this package grants the user flexibility in choosing what information they want to convey, as some plots are more appropriate for certain purposes. For example in Fig. 1d–f are all showing the exact same set of interactions but the cluster of ligands all interacting with the LAG3 receptor is much more obvious from panel e than the others.

As mentioned above, certain tools for predicting cell–cell interactions from single-cell RNA-sequencing data also provide functions for visualizing the results [e.g. CellPhoneDB (Efremova *et al.* 2020) and Liana (Dimitrov *et al.* 2022)]. Many of these visualizations are extremely useful and visually pleasing but it can be difficult to use these plotting functions if a different tool was used for predicting cell-cell interactions. For this reason, the cc_heatmap and cc_dotplot functions in CCPlotR provide an option to reproduce plots in the same style as these popular tools, regardless of which tool was used to generate the results.

All plotting functions included in CCPlotR rely on a default colour palette which is colourblind-friendly and the plots produced are publication-ready. The plots are also easily customizable as all the plotting functions (with the exception of the cc_circos function) return ggplot2 objects, enabling the user to easily modify plots according to their tastes and requirements.

### 2.3 Documentation and availability

The package documentation, along with examples of all the plotting functions, is available on GitHub (https://github.com/Sarah145/CCPlotR). It can be easily installed by running the code chunk below.

if(! require(“devtools”, quietly = TRUE))

     install.packages(“devtools”)

devtools::install_github(“Sarah145/CCPlotR”)

CCPlotR includes a toy dataset (called toy_data) to demonstrate the different functions and provide an example of what the input data should look like. There is also a User Guide vignette included in the package which documents the usage of the different functions.

## 3 Discussion

CCPlotR is an easy to use R package, containing functions that allow users to generate publication-ready graphs of complex data using only a single command. We believe that CCPlotR will provide a useful resource to researchers analysing cell-cell interactions.

## Data Availability

The data associated with this article are openly available in the R package CCPlotR at https://github.com/Sarah145/CCPlotR.
